# A proposed behavioral tool to assess sustained auditory attention

**DOI:** 10.1016/S1808-8694(15)30104-X

**Published:** 2015-10-19

**Authors:** Mariza Ribeiro Feniman, Roberta Ribeiro Ortelan, José Roberto Pereira Lauris, Carolina Ferreira Campos, Mariana Sodário Cruz

**Affiliations:** a Associate Professor - Department of Speech and Hearing Therapy - Schoof of Dentistry Bauru - University of São Paulo.; b Specialist in Clinical Psychology by the Hospital of Craniofacial Anomalies Rehabilitation of São Paulo, HRAC/USP, M.S in Rehabilitation Sciences - HRAC/USP.; c PhD in Sciences/Communications Disorders - HRAC/USP., Professor - Department of Dental-Pediatrics, Orthodontics and Colective Health - Dental School of Bauru - University of São Paulo, FOB/USP.; d M.S. in Pediatrics - Júlio de Mesquita Filho University - UNESP/Botucatu, Speech and Hearing Therapist. MS. Student in Colective Health - Department of Publich Health - Medical School of Botucatu. Department of Speech and Hearing Therapy - Dental School of Bauru - University of São Paulo.

**Keywords:** attention, evaluation, behavioral, children, ability

## Abstract

Sustained attention and vigilance are processeses that characterize attention, and are essential for the development of certain abilities

**Aim:**

a prospective study to propose a simple, easy and quick behavioral tool to support the assessment of sustained auditory attention.

**Material and methods:**

volunteer children aged between 6 and 11 years (139 female and 141 male) were selected. The test was named Sustained Auditory Attention Ability Test, and is based on the Continuous Performance Test. It consist of a binaural and diotic presentation of a list of 100 monosyllabic words in which a target monosyllable is included 20 times. This list was presented six times with no breaks. The test was carried out in a soundproofed room, using a CD player attached to a two-channel audiometer at 50 dBSL during 9 minutes. The test resulted in a total score and a vigilance decrement.

**Results:**

There was no statistically significant difference between genders, but a significant difference was found between ages.

**Conclusion:**

The proposed test had no discomfort for the participants, and was shown to be extremely promising to assess the sustained auditory attention ability in children.

## INTRODUCTION

Attention may be characterized by its selectivity and intensity. Selectivity narrows the focus of information processing from a broad range of stimuli, thoughts and answers, to a simple aspect in the environment, or a selected group of stimulus-response activities. Intensity improves information processing quality, since information processing focus is reduced. This results in an improvement in the quality of cognitive activities involved in the attention behavior. This last aspect is called sustained attention.^1^

One of the most popular ways to assess sustained attention is the Continuous Performance Test-CPT), which requires the individual to keep awake and react to the presence or absence of a target stimulus that has been previously specified. It has numerous presentation methods (auditory, visual or verbal). Having in mind that this test requires the skill to focus and sustain attention during the entire task, default errors (lack of attention) may happen when attention falls and the individual fails in responding to the target stimulus. Impulsiveness-related mistakes happen when an answer is given in the absence of such stimulus.^2^, ^3^

Medical literature^4^, ^5^, ^6^, ^7^, ^8^ has shown that measures using continuous performance tasks have been giving their contribution in the investigation of sustained attention skills in different populations. Nonetheless, in our national reality, the use of these tasks, as well as these behavioral tests that specifically assess this important skill have been proven necessary due to its scarcity.

The present investigation aims at proposing a simple behavioral instrument of easy and fast application in order to assess the capacity of sustained auditory attention.

## MATERIALS AND METHODS

This study was approved by the Ethics in Research Committee under protocol # 093/2004-UEP-CEP. The study was carried out in 2004.

In this study, 280 volunteer Brazilian children participated, 141 males and 139 females, in the age range between 6 and 11 years. All the children had normal peripheral hearing. They did not have auditory complaints and/or upper airways disorder at the time of the investigation, nor prior history of lack of attention and any other difficulty to understand the tests.

Table 1 presents the distribution of the children sampled according to age, gender and their corresponding percentage in the series.

**Table 1:** Distribution of the number of children according to gender and age.

AGE (years)	GENDER		
	M	F	TOTAL
6	21	26	47 (16,7)
7	21	19	40 (14,2)
8	24	33	57 (20,3)
9	20	30	50 (17,8)
10	25	21	46 (16,4)
11	28	12	40 (14,2)
TOTAL	139	141	280 (100)

The entire group sampled underwent the test intended to assess sustained auditory attention capacity (SAAC).

SAAC 9 is based on the ACPT-Auditory Continuous Performance Test5, which is clinically employed to measure auditory attention.7

SAAC is performed in an acoustically prepared booth, with the support of a CD player coupled to a two channel audiometer at an intensity of 50 dBSL, considering the average of the auditory air thresholds for each ear, presented to both ears, in a diotic fashion, with average duration of 9 minutes.

SAAC is a method of objective information used to describe children’s auditory attention behavior. It is used to assess auditory attention by evaluating the child’s capacity to hear auditory stimuli during a prolonged period of time and respond to one specific stimulus only. It is a task of auditory surveillance, indicated by the correct answers to specific linguistic clues, and to measure sustained attention indicated by the child’s capacity to keep attentive and focused on the task for a prolonged period of time. It is based on the presentation, by means of ear phones, of a list of 21 monosyllabic words, recorded by a male voice and presented at the rate of one word per second, which are repeated and randomly rearranged, making up a list of 100 words, including the 20 occurrences of the target word “no”, randomly arranged. This list (recorded in a CD) is presented six times, without interruption.

The 21 monosyllabic words were obtained from a pilot study previously carried out with 43 children between 6 and 7 years of age (average of 6 years and 2 months) who went to public schools in the municipality. These monosyllable words were selected because they are used daily and are reported by the children as being of easy understanding; and the word “no” was reported as being the most easily identifiable, thus being chosen as “target word” of our test. We were careful as to make the other monosyllable words in the list not phonetically close to the word “no”, so that the errors made in the test were related to attention only, and would not have any interference of the difficulties in sound discrimination.

The 21 words selected were: no (target word), foot, yes, flower, goal, train, sea, sun, want, mine, salt, dad, gas, will, sky, now, powder, and one (words which are monosyllables when uttered in Portuguese).

The child was verbally instructed that he/she would hear a list of words and that they should raise the hand whenever the word “no” was heard.

Before the first presentation of the list containing the 100 words of the SAAC test was present to the child, a sample recorded in a CD, of 50 monosyllable words was presented without interruption, and 10 of these words were the word “no”. Only after the child truly understood the task, the test was started.

The answers from the children were marked with an X in the response protocol (list of monosyllables), in front of each word of the test to which the child raised the hand.

In order to determine the result of the SAAC test, the errors were counted and we calculated the reduction in attention.

Errors were considered for two types of responses from the children: Lack of attention: when the child raised the hand in response to the target-word “no” before the next word was introduced; error caused by impulsiveness: when the child raised his or her hand for another word instead of the word “no”.

A count of the number of disattention errors added to the number of impulsiveness errors allowed us to obtain the total score of the SAAC test.

Attention was measured by calculating the number of correct responses to the word “no” for each one of the six presentations. It is necessary to calculate this measure in order to check the reduction in attention span, that is, the reduction in attention that the child suffered during the task, which was obtained by calculating the number of correct responses for the 6th presentation. The difference between these two numbers found is what we call attention span reduction.

Following the goal proposed, the results from the SAAC test were analyzed and calculated for each age in the range between 6 and 11 years and 11 months, and were compared amongst each other. In comparing gender and age, we used the ANOVA variance analysis in two criteria with fixed model.

We used the percentile to check for the prevalence of attention span reduction that was calculated.

## RESULTS

Based on the results obtained in the SAAC test, we created Table 2, that shows the distribution of mean values and standard deviations for the sampled children in each age range, according to gender, considering the lack of attention errors, impulsiveness and total score.

**Tabela 2:** Valores médios (desvios-padrão) dos erros e pontuação total nas faixas etárias segundo os gêneros.

ERROS						
I	Lack of attention		Impulsiveness		Total Score	
	M	F	M	F	M	F
6	25,6 (11,8)	28,7 (16,0)	5,9 (4,1)	8,0 (8,7)	31,5 (13,9)	36,7 (8,7)
7	18,2 (10,6)	22,1 (11,4)	4,2 (3,4)	5,0 (3,2)	22,5 (10,5)	27,1 (3,2)
8	17,1 (11,1)	17,0 (12,3)	3,7 (2,4)	3,7 (3,0)	20,8 (11,6)	20,7 (3,0)
9	12,6 (8,0)	11,8 (8,8)	3,6 (3,5)	5,1 (7,4)	16,2 (9,9)	16,8 (7,4)
10	9,4 (8,3)	10,1 (7,0)	4,0 (3,8)	4,0 (4,3)	13,4 (9,8)	14,1 (4,3)
11	9,0 (9,4)	7,5 (5,5)	2,6 (2,0)	2,0 (2,8)	11,7 (9,8)	9,5 (2,8)

For lack of attention errors, we did not find statistically significant differences between the genders (F = 0.48; p = 0.491) and not also between gender and age (F = 0.42; p = 0.831); however we did notice it between ages (F = 19.46; p = 0.000).

For impulsiveness errors, we did not find statistically significant differences between the genders (F = 1.03; p = 0.311) nor between gender and age interaction (F = 0.53; p = 0.753); however, we did observe it among the ages studied (F = 4.33; p = 0.00).

For total score, we did not find statistically significant differences between the genders for impulsiveness-related errors (F = 1.07; p = 0.301) nor between the gender and age interaction (F = 0.61; p = 0.686), however this difference was observed among the ages studied (F = 22.72; p = 0.000).

**Figure 1:**
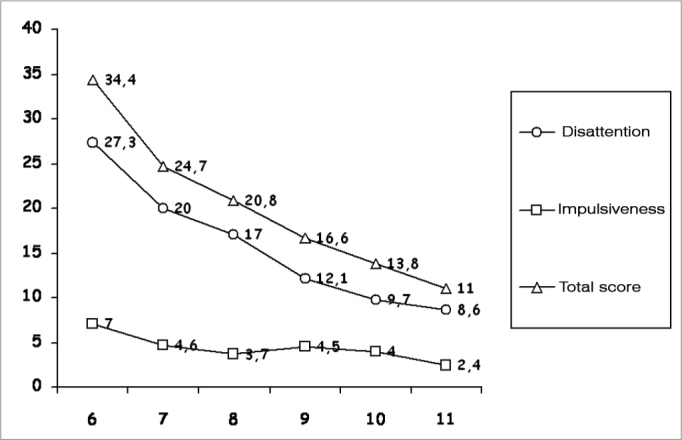
Mean values of the lack of attention errors, of impulsiveness and total score in the age ranges studied for the total group - Legend: I = Age (years) M = Male F = Female.

Considering that there was no statistical difference for both genders for the two types of errors and the total score of the ages studied. Figure 1 shows the distribution of average values considering the entire group sampled.

As to the reduction in attention span, we found the following respective values of 4, 5 and 6 and of 2, 3 and 4 for percentile 75 (25%), percentile 85 (15%) and percentile 90 (10%), respectively for the ages of 6-8 years and 9-11 years. Reductions of 8 and 9 were found in only 5% (P95) and 1% (P99) for the ages of 6-8 years and, of 5 (P95) and 6 (P99) for the ages 9-11 years.

The reduction in attention span found in more than 10% (P90) of the children was the most observed, and values below 10% (P95 and P99) was considered significant and suggestive of a problem in the child’s capacity for sustained auditory attention.

## DISCUSSION

There are numerous characteristics of this study that make it adequate as a proposal for an instrument to assess the sustained auditory attention capacity. First, the scarcity of tools in our country for this specific goal. The series in our study have enough children (40 to 50) in all age groups intended. Except for the age of 11 years, the number of boys and girls was balanced in all age levels, thus providing an opportunity to examine changes in performance development in SAAC.

In regards of the instrument itself, both in the clinic and in the investigation, the use of CPT (Continuous Performance Test), may very well be the most frequently used measure of attention.^10^

When we compare our results with those in the literature^4^, ^5^, ^11^, we notice an agreement for the lack of significance in the variable gender, disagreeing from the paper^12^, of which girls had a worse performance in the rates of correct answers and the test sensitivity for continuous performance, however with lower stimuli. A prior study^13^ shows higher impulsiveness scores for males, since impulsiveness control develops earlier on for females.^14^

The lack of attention errors score seen was inversely proportional to age, that is, higher values attained at younger ages indicate that older children perform better and make fewer mistakes than smaller children, in agreement with the literature.^8^, ^12^, ^15^, ^16^, ^17^ Younger children have a more limited attention span, and as they grow, there are changes in their inner processing mechanisms that increase this capacity.^18^

Younger individuals seem to have a more impulsive behavior than their older counterparts^11^, in accordance with the present investigation.

Any sustained attention task must include the perception of a signal, a memory of it or of a code to determine it and a skill to discriminate what is a signal and what is not.^19^ Many of the tasks that have been used to study attention have short memory requirements, which improves with the child’s development.^20^ Sustained and selective hearing attention tasks require a continuous work memory for this successful performance. Correct answers in this task continually require working memory skills, in which each stimulus has to be stored in the long term memory long enough to be compared to the stimuli that follow.^21^ Thus, based on the score attained in this study, showing higher values than the ones attained in prior studies5, one may think that Brazilian children may have had difficulties in these other skills required for a better sustained attention performance.

There is a study^22^ that reports on lack of attention as a problem that makes the person lose or not record the information in their working memory in order to process it. Thus, these individuals spend more time in their work or school tasks, trying to recover such information that they lost (due to their lack of attention) and, as a result, information processing is delayed.

A child guides and sustains attention when something is of their interest. There is a motivational phenomenon occurring underneath the attention cognitive phenomenon^23^, thus, the task motivation and pleasure may have been insufficient for the younger children studied, caused by the very “boring” nature of the test.^6^ Difficulties with attention may be revealed when a task requires one to keep up the effort in activities of this sort^24^, having in mind that the auditory attention test hereby applied presents continuous and unbroken monosyllabic words, not allowing the stimulus to be repeated.

A reduction in attention may become apparent when a task or test has a time to be finished or requires the child to be alert to receive continuous stimuli. Thus, the child starts to fail, or miss items, anxiety increases and performance declines.^24^ That being, in regards of the duration of the test hereby proposed, it was very close to a prior study.^5^ A long standing task may increases the number of errors caused by impulsiveness.^25^

Children may start a task at a given concentration level, which is not possible to sustain, and consequently there is a decline in performance. Performance drops of the participants during the second half of a twenty minute attention task has been observed in studies.^26^, ^27^ Nonetheless, authors have assigned attention decline to mental fatigue, which they deem is not related to sustained attention.

In general, tasks with faster event proportions produce more errors.^28^ The present investigation used the rate of one word per second. One word per second was more effective than one word every two seconds.^5^

Although a significant attention decline is considered characteristic of individuals with attention deficits, a short decline is common in children of the general population who do not have such deficit.^5^ Consistent with the previous study^5^, data from the present investigation show steeper declines in children between 6 and 8 years when compared to those of 9 to 11 years. This performance improvement in older children results, very likely, from the development of compensatory strategies in order to pay attention to the tasks.^5^

Tasks, environment, participating factors and their interactions may also produce different effects in the distinct performance measures.^28^ However, special care was taken in order to follow the recomendations^5^ as to checking the devices used, the ear phones, their proper functioning and audiometer calibration, the silent place, without distraction or noise that would interfere or mask the responses from children, and we also tried to be face-to-face with the child being assessed, and this allowed us to observe the child’s behavior during the entire test.

And finally, despite the scores obtained in the present study proved to be higher when compared to a previous study^5^, we can see an important behavior similarity among them, leading us to believe that SAAC can be a highly regarded test to assess the child’s capacity to sustain hearing attention.

## CONCLUSION

The test proposed to assess the sustained auditory attention capacity (SAAC), which proved to be of easy and fast application and highly regarded to assess the child’s capacity to sustain auditory attention.
